# Pulvinar Lesions Disrupt Fear-Related Implicit Visual Processing in Hemianopic Patients

**DOI:** 10.3389/fpsyg.2018.02329

**Published:** 2018-11-22

**Authors:** Caterina Bertini, Mattia Pietrelli, Davide Braghittoni, Elisabetta Làdavas

**Affiliations:** ^1^Department of Psychology, University of Bologna, Bologna, Italy; ^2^Centre for Studies and Research in Cognitive Neuroscience, University of Bologna, Cesena, Italy

**Keywords:** hemianopia, affective blindsight, pulvinar, fear, implicit visual processing

## Abstract

The processing of emotional stimuli in the absence of awareness has been widely investigated in patients with lesions to the primary visual pathway since the classical studies on affective blindsight. In addition, recent evidence has shown that in hemianopic patients without blindsight only unseen fearful faces can be implicitly processed, inducing enhanced visual encoding (Cecere et al., 2014) and response facilitation (Bertini et al., 2013, 2017) to stimuli presented in their intact field. This fear-specific facilitation has been suggested to be mediated by activity in the spared visual subcortical pathway, comprising the superior colliculus (SC), the pulvinar and the amygdala. This suggests that the pulvinar might represent a critical relay structure, conveying threat-related visual information through the subcortical visual circuit. To test this hypothesis, hemianopic patients, with or without pulvinar lesions, performed a go/no-go task in which they had to discriminate simple visual stimuli, consisting in Gabor patches, displayed in their intact visual field, during the simultaneous presentation of faces with fearful, happy, and neutral expressions in their blind visual field. In line with previous evidence, hemianopic patients without pulvinar lesions showed response facilitation to stimuli displayed in the intact field, only while concurrent fearful faces were shown in their blind field. In contrast, no facilitatory effect was found in hemianopic patients with lesions of the pulvinar. These findings reveal that pulvinar lesions disrupt the implicit visual processing of fearful stimuli in hemianopic patients, therefore suggesting a pivotal role of this structure in relaying fear-related visual information from the SC to the amygdala.

## Introduction

The ability to extract emotional information from facial expressions is crucial for successful adaptation in social environment. Due to its importance for survival, this ability seems to be preserved also in the absence of awareness (for a review, Tamietto and de Gelder, 2010; Celeghin et al., 2015; Diano et al., 2017). In line, the studies investigating the peculiar phenomenon of affective blindsight have shown that patients with lesions of the primary visual cortex (V1) can unconsciously perceive emotional signals, demonstrating performance above chance when guessing the emotional content of faces shown in their blind field, in forced choice tasks (de Gelder et al., 1999, 2001). In addition, recent studies have revealed the presence of implicit emotional processing also in hemianopic patients without any form of blindsight or affective blindsight (Bertini et al., 2013, 2017; Cecere et al., 2014). In these studies, patients with visual field defects, who perform at the chance level in discriminating the emotional content of stimuli in their blind field in forced choice tasks, have shown behavioral and electrophysiological evidence of implicit processing of unseen fearful stimuli. Specifically, when hemianopic patients were required to respond to faces displayed in their intact field, while emotional faces were simultaneously presented in their blind field, they showed a reduction of response time (i.e., a response facilitation) only when fearful faces were concurrently displayed in their blind visual field (Bertini et al., 2013). In contrast, no facilitation was found during the concurrent presentation of unseen happy or neutral faces (Bertini et al., 2013). In addition, the presentation of fearful faces in the blind field has been shown to increase the amplitude of the electrophysiological component N170, evoked by faces presented in the intact field, therefore suggesting an enhancement of the visual structural encoding of seen faces, occurring at the early stages of visual processing (Cecere et al., 2014). Similarly, a recent study has also demonstrated that the facilitatory effects of unseen fearful faces can generalize outside the facial domain, showing a reduction of response time to simple visual stimuli (Gabor patches) displayed in the intact field (Bertini et al., 2017). Overall, these findings suggest that when a lesion occurs to the cortical visual pathway, fear-related visual information in the blind visual field can be extracted in the absence of awareness, improving visual processing in the intact visual field.

This implicit visual processing for unseen threat-related information has been suggested to be mediated by a subcortical pathway from the superior colliculus (SC) to the amygdala, via the pulvinar nuclei of the thalamus (LeDoux, 1996). In line, the structures encompassing this circuit have demonstrated enhanced positive covariation of activity to unconsciously perceived emotional expressions (Morris et al., 1999; Liddell et al., 2005).

The specificity of this pathway for rapid visual processing of socially relevant stimuli, such as faces, has been reported in studies on primates, demonstrating that the neurons in the superficial layers of the SC show early responses (firing ∼25–50 ms after stimulus onset) to facial information (Rizzolatti et al., 1980; Nguyen et al., 2014, 2016). In addition, neurons in the dorsal lateral pulvinar and the ventral part of the medial pulvinar have shown responses to face and face-like stimuli with latencies <60 ms (Nguyen et al., 2013). Notably, pulvinar neurons also showed differential responses to facial expressions (Maior et al., 2010). Finally, both human intracranial (Méndez-Bértolo et al., 2016) and MEG data (Luo et al., 2007) have revealed early responses to faces expressing fear in the amygdala occurring with latencies lower than 75 ms after stimulus-onset. Importantly, the existence of direct connections between these anatomical structures has been supported by neurophysiological evidence on rats (Day-Brown et al., 2010) and research using diffusion tractography in both monkeys and humans (Tamietto et al., 2012; Rafal et al., 2015; Koller et al., 2018).

These converging findings propose the pulvinar as a crucial connectional hub of the subcortical pathway mediating fear-related visual processing in the absence of awareness. In line with this reasoning, previous evidence on hemianopic patients have demonstrated the relevance of the pulvinar also in mediating implicit visual processing of motion stimuli: indeed, while hemianopics without pulvinar lesions showed enhanced BOLD responses, in hemianopics with lesions involving the pulvinar no activity was observed after the presentation of motion stimuli in their blind field (Barleben et al., 2015), thus corroborating the relevance of this subcortical structure in mediating visual processing for relevant stimuli in the absence of awareness. Therefore, it might be hypothesized that in the presence of lesions to the pulvinar, also the facilitatory effects due to implicit visual processing of fearful faces should not be evident. In order to test this hypothesis, hemianopic patients without blindsight, with or without pulvinar lesions, were required to discriminate the orientation of Gabor patches displayed in their intact visual field, while fearful, happy or neutral faces were simultaneously shown in their blind field. In line with previous evidence (Bertini et al., 2017), hemianopic patients without pulvinar lesions are expected to show reduced response times to stimuli in the intact field, only when fearful faces are displayed in the blind visual field. In contrast, no response facilitation is expected in hemianopic patients with pulvinar lesions, therefore suggesting a prominent role of this subcortical structure in relaying fear-related visual information from the SC to the amygdala.

## Materials and Methods

### Participants

Twelve patients with right visual field defects, as documented by an automated perimetry test, participated in Experiments 1 and 2. All patients were right-handed and had corrected-to-normal or normal visual acuity. In addition, no concurrent psychiatric or neurological disorders or cognitive deficits were present. After being informed about the procedure, all patients provided written informed consent to participate. The study was approved by the Ethics Committee of the Department of Psychology of the University of Bologna, according to the ethical principles of the World Medical Association Declaration of Helsinki.

**FIGURE 1:**
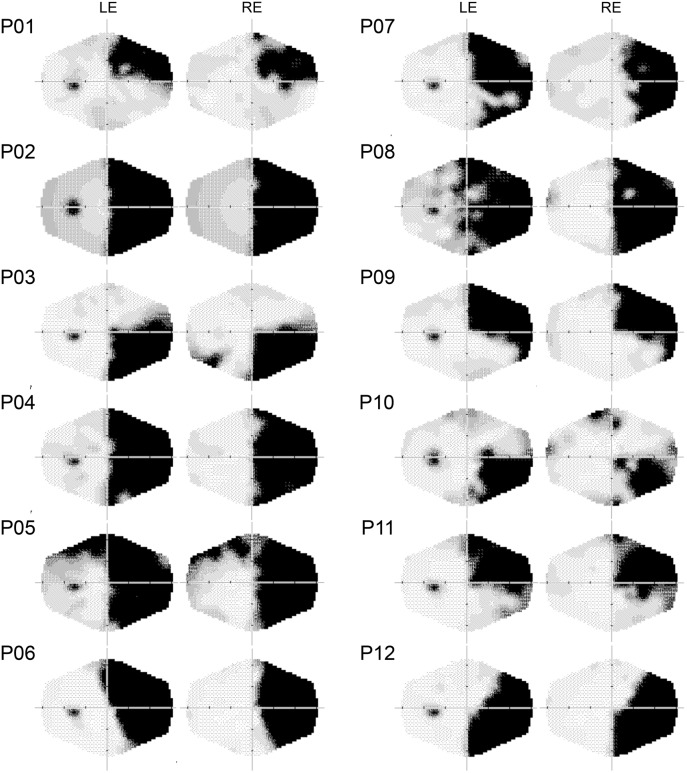
Computerized automated visual perimetry (Medmont M700 automated perimetry apparatus, Melbourne, VIC, Australia). Axial hash marks denote 10 visual degree increments. LE, left eye; RE, right eye.

**FIGURE 2:**
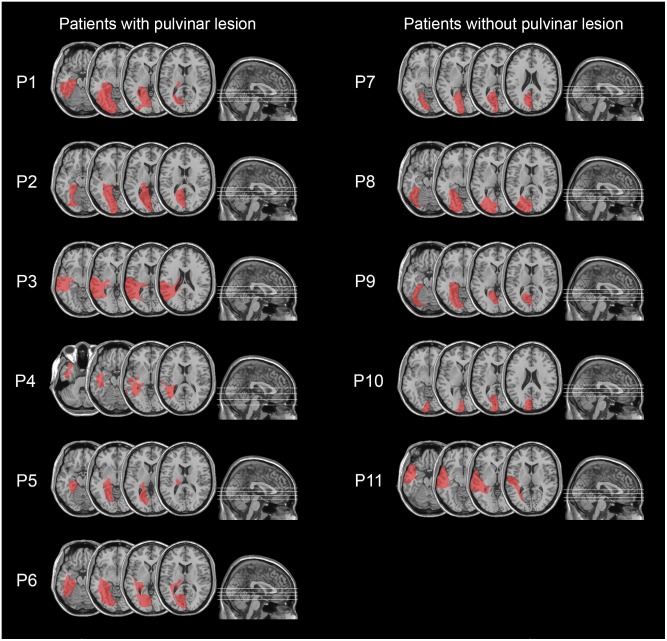
Lesion reconstruction images from CT or MRI scans, projected onto the normalized MNI template for hemianopic patients with pulvinar lesions (P1–P6; left column) and hemianopic patients without pulvinar lesions (P7–P11; right column).

**FIGURE 3:**
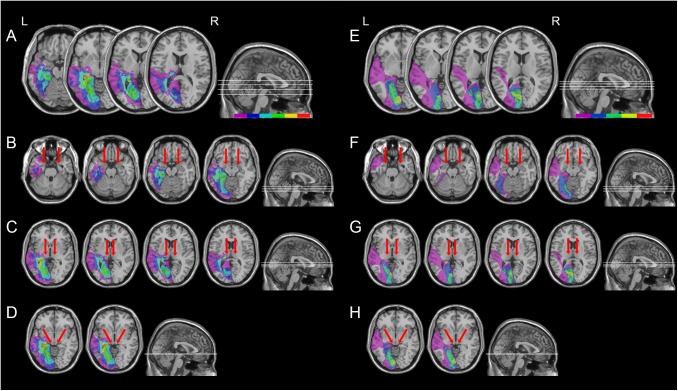
Location and overlap of brain lesions of hemianopic patients with or without pulvinar lesions. The image shows the lesions of the hemianopic patients with pulvinar lesions **(A)** and hemianopic patients without pulvinar lesions **(E)** projected onto four axial slices of the standard MNI brain. In each slice, the left hemisphere is on the left side. The levels of the axial slices are marked by white lines on the sagittal view of the brain. The color bar indicates the number of overlapping lesions. Panels **B–D** show overlap of the lesions of hemianopic patients with pulvinar lesions projected onto the axial slices where the amygdala **(B)**, the pulvinar **(C)**, and the superior colliculus **(D)** are visible. Panels **F–H** show overlap of the lesions of hemianopic patients without pulvinar lesions projected onto the axial slices where the amygdala **(F)**, the pulvinar **(G)**, and the superior colliculus **(H)** are visible. The arrows indicate the amygdala **(B,F)**, the pulvinar **(C,G)**, and the superior colliculus **(D,H)**.

All patients had post-geniculate lesions in the left hemisphere, resulting in deafferentation or damage of the striate cortex, documented by magnetic resonance imaging (MRI) or computed tomography (CT). Six patients had additional pulvinar lesions (1 female; *M* age = 54.8 years; *M* education = 11.7 years; *M* time since lesion onset = 27 months), while in the other six patients the pulvinar was spared (1 female; *M* age = 49.5 years; *M* education = 12.2 years; *M* time since lesion onset = 9.6 months; Table 1 and Figures 1, 2). Brain lesions were mapped using MRIcro (Rorden and Brett, 2000; Rorden et al., 2007), based on the most recent clinical CT or MRI. Although manual lesion tracing procedures have the limit to rely greatly on anatomical expertise, and to be subjective in nature, they circumvent problems frequently encountered by automated normalization procedures. Indeed, while automated procedures have greatly improved (Clas et al., 2012; Rorden et al., 2012; Zhang et al., 2014; de Haan et al., 2015; Pustina et al., 2016), variation in clinical image quality, which might be due to the nature of the imaging protocol, the quality of the imaging hardware and differences in head movement, might prevent automatic normalization into a standard template (Kimberg et al., 2007). Therefore, lesions were manually traced onto the T1-weighted MRI template provided with MRIcro software (with the exception of P12, whose MRI scans were not available; Rorden and Brett, 2000; Rorden et al., 2007). The number of damaged voxels was calculated for each patient and the lesion volumes were compared between the two groups. No significant differences were found in lesion volumes between hemianopic patients with additional pulvinar lesions (70188 mm^3^; Figure 3A) and hemianopic patients without pulvinar lesions [47915 mm^3^; *t*(9) = 1.24; *p* = 0.25; Figure 3E]. As shown by overlaps of brain lesions in Figure 3, in patients with pulvinar lesions, the superior colliculus and the amygdala were spared (Figures 3B–D). Patients without pulvinar lesions reported damage to brain areas not including the amygdala, pulvinar, and superior colliculus (Figures 3F–H). No differences between the two groups were found relative to time since lesion onset [*t*(10) = 1.79; *p* = 0.1], age [*t*(10) = −0.61; *p* = 0.55] or education [*t*(10) = −0.21; *p* = 0.84]. Clinical details are reported in Table 1.

**Table 1:** Summary of clinical, demographic, and lesional data.

Case	Sex	Age	Years of education	Time since lesion onset (months)	Visual field defect	Etiology	Cortical lesion site
P1	M	71	13	6	Right superior quadrantanopia	Vascular	Left temporal-occipital
P2	M	39	13	3	Right hemianopia	Vascular	Left occipital
P3	F	38	18	33	Right inferior quadrantanopia	Vascular	Left frontal-temporal-parietal
P4	M	45	13	42	Right hemianopia	Vascular	Left temporal-parietal
P5	M	81	5	18	Right hemianopia	Vascular	Left temporal-occipital
P6	M	55	8	60	Right superior quadrantanopia	Vascular	Left temporal-occipital
P7	M	57	13	5	Right hemianopia	Vascular	Left occipital
P8	F	32	18	4	Right hemianopia	Vascular	Left temporal-parietal-occipital
P9	M	50	13	15	Right superior quadrantanopia	Vascular	Left temporal-parietal-occipital
P10	M	65	8	5	Right inferior quadrantanopia	Vascular	Left occipital
P11	M	52	8	25	Right hemianopia	Traumatic	Left temporal
P12	M	41	13	4	Right inferior quadrantanopia	Vascular	Left occipital

*M, male; F, female.*

### Procedure

Experiments 1 and 2 were performed in a sound attenuated room with dimmed light. Patients sat at a distance of 57 cm, in front of a 17″ LCD monitor (refresh rate: 60 Hz). A Pan/Tilt optic eye-tracker (Eye-Track ASL-6000; sampling rate 60 Hz) monitored eye movements. Presentation software^1^ (version 0.60) controlled stimulus presentation and recorded responses. Patients were required to fixate a central white cross (2°), avoiding eye movements. For patients P1, P6, and P9 (right superior quadrantanopia) and P3, P10, and P12 (right inferior quadrantanopia), the fixation cross was horizontally centered, on either the upper or the lower edge of the monitor (2° from the edge), to ensure stimuli were presented in the blind quadrant.

#### Experiment 1: Two-Alternative Forced Choice Tasks

To make sure that hemianopic patients with or without pulvinar lesions showed no sign of blindsight, they performed a two-alternative forced choice (2AFC) task, testing different stimuli in four separate sessions. We used the same experimental paradigm used in previously published studies (for details, see Bertini et al., 2013, 2017). Stimuli were only shown in their blind visual field, while no stimuli were presented in their intact visual field. Each stimulus was presented at 10° of eccentricity on the horizontal plane (either to the left or to the right of the central fixation cross, based on the side of hemianopia). For patients with upper quadrantanopia, the fixation cross was placed at the lower edge of the monitor, while for patients with lower quadrantanopia it was placed at the upper edge of the monitor, to ensure that stimuli were presented in the blind quadrant. The stimuli and the central fixation cross were presented on a gray background. In the visual detection task, a white dot (2° diameter) was used as stimulus. In the emotional task, emotional faces consisting of grayscale photographs (7° × 5°) of six different actors (three males), with happy or fearful expressions, were used as stimuli (Ekman and Friesen, 1976). In the gender task, the stimulus consisted of grayscale photographs (7° × 5°) of different faces (three males) showing a neutral expression (Ekman and Friesen, 1976). In each photograph of both the emotional and the gender task the hairline was removed using Adobe Photoshop. In the geometrical shapes task, stimuli consisted of white circles and squares (5° × 5°). At the beginning of each trial, a central fixation cross (500 ms) was presented. Subsequently, the target stimulus, if present, was displayed for a duration of 1500 ms. After the presentation of each stimulus, a fixation cross appeared again (250 ms; total trial duration: 2250 ms) and a sound prompted patients to verbally respond. The experimenter manually recorded patients’ verbal responses. After the response, the experimenter, monitoring patients’ eye position, manually started a new trial, only when their gaze was on the fixation cross. Trials contaminated with eye movements were removed (0.5%). Stimuli were presented in a random order. When performing the visual detection task, hemianopics were instructed to decide whether or not the stimulus was shown in the blind visual field. In the emotional, gender and shape tasks, they were asked to guess, choosing between two alternatives, which sort of stimuli was shown in their blind field. More precisely, in the emotional task, they had to discriminate happy or fearful faces, in the gender task they were asked to discriminate female or male faces, while in the geometrical shapes task they had to discriminate square or circle. The order of the 2AFC tasks was counterbalanced between participants. One hundred and eighty trials were presented in each 2AFC task (90 trials of each of the two possible alternatives). The percentage of correct choices was calculated in each task, for each patient. A Binomial test was used to compare the accuracy to the chance level (50% correct choices).

#### Experiment 2: Go/No-Go Task With Redundant Stimuli

Patients were tested with a go/no-go task, in which stimuli were presented concurrently in the blind and the intact visual field, exploiting the same experimental paradigm used in previously published studies (for details, see Bertini et al., 2017). Target stimuli were shown in the intact field and were paired with concurrently presented stimuli in the blind field. Concurrent stimuli were presented in a random order 10° to the left and to the right of the center of the monitor on the horizontal plane. For patients with upper quadrantanopia, the fixation cross was placed at the lower edge of the monitor, while for patients with lower quadrantanopia it was placed at the upper edge of the monitor, to ensure that stimuli contralateral to the lesion were presented in the blind quadrant. The stimuli and the central fixation cross were presented on a gray background. Gabor patches (diameter: 2°; spatial frequency: 8 Hz) were used as target stimuli and were created with Matlab (The MathWorks Inc., Natick, MA, United States). The Gabor patches were displayed in patients’ intact visual field, while emotional faces were simultaneously presented in their blind field. Emotional faces consisted of 18 grayscale photographs (7° × 5°) of six different actors (three males) displaying fearful, happy, or neutral expressions (Ekman and Friesen, 1976). In each photograph, the hairline was removed using Adobe Photoshop. At the beginning of each trial, a fixation cross (500 ms) appeared. Then, the pairs of stimuli were displayed for a duration of 200 ms, followed by a blank screen (1000 ms). After a random inter-trial interval (500–800 ms), a new trial automatically started. Trials contaminated with eye movements were removed (2%). Patients performed a total of six blocks of the go/no-go task with redundant stimuli. In three blocks, they were required to provide rapid responses (by pressing the spacebar on a keyboard) to Gabor patches with a horizontal orientation and to avoid response to Gabor patches with a vertical orientation; in the remaining three blocks, the response requirements were reversed. They performed a total of 216 trials (in half of the trials the horizontal Gabor patch was the target: 54 trials for the target/distractor stimuli – 18 for each of the three unseen emotional faces-; in the remaining half of the trials the vertical Gabor patch was the target: 54 trials for the target/distractor stimuli – 18 for each of the three unseen emotional faces-). Response times more than two standard deviations below or above the mean were discarded (4.5%), to control for outliers. The responses to vertical and horizontal Gabor patches were collapsed. We analyzed response times and the percentage of correct responses with two analyses of variance (ANOVAs) with Group (hemianopic patients WITH pulvinar lesions, hemianopic patients WITHOUT pulvinar lesions), as between-group factor, and Condition (unseen fearful faces, unseen happy faces, and unseen neutral faces), as within-group factor. Newman–Keuls test was used for *post hoc* comparisons.

## Results

### Experiment 1: Two-Alternative Forced Choice Tasks

Individual performance of patients with or without pulvinar lesions did not significantly differ from chance in any of 2AFC tasks (percentages of correct answers are reported in Table 2). Specifically, in the visual detection task, no significant difference from the chance level was found (all *p*s > 0.18). No significant difference form the chance level was also found in performance in the remaining 2AFC tasks: emotional task (all *p*s > 0.18), gender task (all *p*s > 0.18), geometrical shapes task (all *p*s > 0.1). This provide evidence that hemianopics with or without pulvinar lesions had no form of blindsight, showing no awareness for the presence or the nature of unseen stimuli, displayed in their blind visual field.

**Table 2:** Percentages of correct answers in the two-alternative forced choice tasks.

Case	Visual detection task	Emotional task	Gender task	Shape task
P1	49%	52%	52%	54%
P2	52%	47%	46%	53%
P3	48%	49%	53%	51%
P4	51%	54%	51%	48%
P5	54%	52%	48%	52%
P6	47%	53%	45%	54%
P7	55%	45%	51%	44%
P8	46%	49%	52%	53%
P9	52%	53%	53%	49%
P10	47%	55%	49%	52%
P11	48%	48%	46%	50%
P12	52%	50%	48%	54%

### Experiment 2: Go/No-Go Task With Redundant Stimuli

**FIGURE 4:**
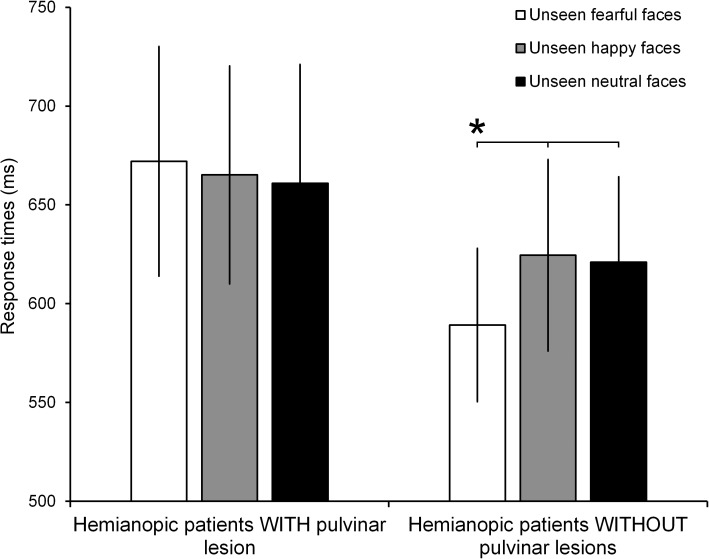
Mean RTs for each condition (unseen fearful faces, unseen happy faces, and unseen neutral faces) in patients with and without pulvinar lesions. Error bars represent the standard error of the mean (SEM). Asterisks indicate a *p* < 0.05.

The ANOVA on the response times to Gabor patches displayed in the intact visual field showed no significant effect of Group (*F*_1,10_ = 0.58, *p* = 0.47; $\eta_{p}^{2}$ = 0.05) or Condition (*F*_2,20_ = 1.50, *p* = 0.25; $\eta_{p}^{2}$ = 0.13). On the contrary, the ANOVA reveled a significant Group × Condition interaction (*F*_2,20_ = 4.18, *p* = 0.03; $\eta_{p}^{2}$ = 0.29). The results of the *post hoc* test showed, in hemianopic patients without pulvinar lesions, a significant reduction of response times to seen Gabor patches paired with unseen fearful faces (589 ms), compared to the conditions in which they were paired with unseen happy (624 ms; *p* = 0.02) or neutral faces (621 ms; *p* = 0.02; Figure 4). Response times in the happy and neutral conditions revealed no significant difference (*p* = 0.78). In contrast, in hemianopics with pulvinar lesions, response times to targets were not modulated by unseen stimuli. Indeed, the response times to Gabor patches in the intact field, paired with simultaneous unseen fearful faces (672 ms) did not show significant differences compared to response times to seen targets, paired with simultaneous happy (665 ms; *p* = 0.58) and neutral faces (661 ms; *p* = 0.63). Again, these two latter conditions revealed no significant difference (*p* = 0.73; see Figure 4).

Results of the ANOVA on the percentage of correct responses showed no significant main effect or interaction (all *p*s > 0.1; mean percentage of correct responses = 89% ± 7%).

## Discussion

Hemianopic patients without blindsight with pulvinar lesions do not show implicit visual processing for fearful faces, in contrast with hemianopics with lesions not involving the pulvinar. Indeed, in keeping with previous studies (Bertini et al., 2017), hemianopic patients without blindsight and whose lesions do not encompass the pulvinar show response facilitation to Gabor patches displayed in their intact visual field, during the simultaneous presentation of faces expressing fear in their blind visual field, but not happy or neutral faces. In contrast, in hemianopic patients with pulvinar lesions, no response facilitation for stimuli presented in the intact field was found.

The fear-specific facilitation in hemianopic patients without blindsight, in which the pulvinar is spared, suggests that, after damage to the cortical visual route, only threat-related visual information can be processed in the absence of awareness. This is in line with previous findings on hemianopic patients without blindsight, showing enhanced visual encoding (Cecere et al., 2014) and response facilitation (Bertini et al., 2013, 2017) to stimuli presented in their intact field, only when fearful faces were displayed at the same time in their blind field. Similarly, a specific implicit visual processing for fearful stimuli has been shown also using fear conditioned neutral faces in patients with visual field defects, corroborating the hypothesis that salient and ecologically relevant stimuli might receive a preferential processing in the absence of awareness (Anders et al., 2004, 2009). This effect has been attributed to the subcortical colliculus-pulvinar-amygdala circuit, spared after the lesion (Bertini et al., 2016). Indeed, this subcortical circuit seems pivotal for the rapid, coarse, and unconscious processing of salient and emotional visual stimuli (for a review: Tamietto and de Gelder, 2010; Garrido et al., 2012; Garvert et al., 2014; McFadyen et al., 2017). In this perspective, the processing of fearful stimuli in the absence of awareness seems to represent an adaptive mechanism, in which the fear-related signals processed by the subcortical circuit might indicate a potential threat, facilitating visual processing in the intact visual field and thus ensuring rapid visual analysis of the surroundings. Alternatively, the observed facilitation in the presence of unseen fearful stimuli might depend also on influences of the subcortical circuit on interconnected motor cortices, which might facilitate the motor response to stimuli toward the intact field. In keeping, a large body of evidence has shown that fearful stimuli alter the state of the motor cortex (de Gelder et al., 2004; Schutter et al., 2008; Borgomaneri et al., 2015, 2017), albeit findings of effects of unconsciously perceived fear on the motor system are rather sparse (Engelen et al., 2018). However, previous EEG investigations on hemianopics without blindsight (Cecere et al., 2014) have shown that the implicit visual processing of fearful stimuli affect the stage of structural encoding of the visual stimuli in the intact field (i.e., the N170 component), therefore suggesting that unconscious fear has influences on the early visual process.

The present results add to previous data by demonstrating that the pulvinar represents a critical relay structure of this subcortical pathway, conveying threat-related visual information from the SC to the amygdala, in the absence of awareness. The pulvinar has been extensively reported to assist in shifting to relevant visual stimuli (Benevento and Miller, 1981; Benevento and Port, 1995; Arend et al., 2008), therefore supporting its pivotal role in the processing of salient visual information. Most knowledge of the importance of the pulvinar in the rapid processing of visual threat has been obtained from primates and humans with selective pulvinar lesions. In monkeys, medial and dorsolateral pulvinar neurons revealed selective responses to snakes, showing larger mean response magnitude and shorter latencies, than responses to other stimuli, therefore suggesting a mechanism facilitating rapid visual detection of fear-relevant stimuli (Van Le et al., 2013, 2014). Evidence on patients has shown that unilateral pulvinar lesions impair discrimination of fearful faces in the visual field contralateral to the lesion (Ward et al., 2007) and undermine the fast processing of threatening stimuli (Ward et al., 2005). In addition, patients with lesions to the pulvinar also demonstrated reduced attentional effects of salient distracter (Snow et al., 2009). In line, increasingly converging evidence from neuroimaging studies show pulvinar activation in the presence of threatening stimuli (Almeida et al., 2015) and fearful facial expressions (Vuilleumier et al., 2003).

The prominent role of the pulvinar as a convergence point for transmitting ascending visual information to the amygdala seems to account for its relevance in fear-related processing (Bridge et al., 2016). Studies on animals have reported that the superficial layers of the SC send visual information to the intermediate and deep layers of the SC (May, 2006; Stein et al., 2009), which, in turn, project to the medial subdivision of the pulvinar (Benevento and Fallon, 1975; Linke et al., 1999; Grieve et al., 2000). Importantly, the medial pulvinar has reciprocal connections with the amygdala (Grieve et al., 2000; Shipp, 2003). Although most of the evidence on the connectivity patterns of the pulvinar arises from anatomical studies on non-human primates, recent tractography studies have demonstrated direct connectivity between the SC, the pulvinar and the amygdala also in humans (Tamietto et al., 2012; Rafal et al., 2015; Koller et al., 2018). Specifically, the fibers connecting these structures ascend from the SC, pass through the medial pulvinar to the pole of the pulvinar, and then descend to the lateral pulvinar to finally connect to the amygdala (Rafal et al., 2015; Koller et al., 2018). Crucially, these fibers are spared and reportedly strengthened after lesions to the visual cortex in patients with affective blindsight (Tamietto et al., 2012), providing further evidence that these connections might represent the anatomical circuit subserving implicit emotional processing. However, it is worth noting that the performance of patients with affective blindsight is different from the performance of hemianopic patients without blindsight in this and in previous studies (Anders et al., 2004, 2009; Bertini et al., 2013, 2017; Cecere et al., 2014). Indeed, affective blindsight patients show above-chance discrimination of emotional faces in forced choice tasks and response facilitation to emotionally–congruent pairs of facial stimuli (de Gelder et al., 1999, 2001; Pegna et al., 2005), regardless the type of emotion. On the contrary, hemianopics without blindsight show fear-specific response facilitation (Anders et al., 2004, 2009, Bertini et al., 2013, 2017; Cecere et al., 2014). Although the subcortical colliculus-pulvinar-amygdala visual pathway seems to contribute to the implicit emotional processing in both patients with affective blindsight and hemianopics without blindsight, their distinct patterns of performance might be attributed to different neural substrates. More precisely, we can speculate that the performance of affective blindsight patients might depend on the contribution of spared and functionally reorganized visual cortices. Such a peculiar functional reorganization might have different accounts, depending both on the etiology or the site of patients’ lesions.

For instance, in the case of the most extensively studied patient with affective blindsight, i.e., G.Y. such a functional reorganization might be the result of plastic changes occurring due to the early onset of his lesion (Celeghin et al., 2015), possibly involving also interhemispheric contributions (Celeghin et al., 2017, 2018). Another well documented case, i.e., patient D.B., with implicit visual processing for a variety of visual features (Weiskrantz, 1986), including the emotional content (de Gelder et al., 2002; Tamietto et al., 2009), underwent surgical removal of a benign tumor at the age of 30, but suffered from visual symptoms from his teens (Weiskrantz, 1986). The slow growth of low-grade benign tumors is known to promote profound plastic changes and, therefore, might account for his peculiar residual abilities (Duffau, 2017). Finally, affective blindsight has been mainly reported in a series of single case studies investigating patients with cortical blindness following bilateral occipital disruption (e.g., Pegna et al., 2005; Solcà et al., 2015; Burra et al., 2017; Striemer et al., 2017). In these patients, the disruption of both visual cortices might have induced a more radical reorganization of the visual pathways conveying visual information from the subcortical structures to the cortex, thus promoting the emergence of their striking visual residual abilities. Overall, although the functional neuroanatomy of the affective blindsight still remain elusive, post-lesional plastic changes occurring to the subcortical V1-independent pathways and their multiple connections with extrastriate areas, both within the dorsal and the ventral stream (Tamietto and Morrone, 2016), might represent a plausible account for this phenomenon. In this perspective, it has been recently proposed that in affective blindsight patients, facial emotional visual information is conveyed from the SC to the pulvinar, from which it is directly projected to extrastriate and temporal cortices, such as the superior temporal sulcus, to finally reach the amygdala (Gerbella et al., 2017). This suggests a significant contribution of extrastriate areas in mediating the above chance performance in discriminating emotional faces and the facilitatory effects for congruent pairs of emotional stimuli, typical of patients with affective blindsight.

In contrast, the fear-specific implicit visual processing observed in hemianopics without blindsight might be subserved only by activity in the subcortical colliculus-pulvinar-amygdala circuit. Behavioral evidence on healthy participants tested with backward-masked emotional faces have provided support to this hypothesis (Cecere et al., 2013). Indeed, participants exhibited fear-specific facilitatory effects (resembling the ones observed in hemianopics) when the activity of the occipital cortex was temporary inhibited by transcranial direct current stimulation (tDCS). In contrast, when tDCS was delivered to a control area and, thus, the activity in visual cortices was not suppressed, congruency-dependent response facilitation (resembling the one observed in blindsight patients) was found (Cecere et al., 2013). However, further studies investigating the fiber tracts spared in hemianopics without blindsight are needed to disentangle the additional possible contribution of subcortical-cortical connections (Tamietto and Morrone, 2016) in mediating fear-specific implicit visual processing. In the present study, only hemianopic patients with left hemispheric lesions were tested, since previous evidence have shown that hemianopic patients with lesions to right hemisphere do not demonstrate the facilitatory effects due to the implicit visual processing of emotional stimuli (Cecere et al., 2014; Bertini et al., 2017). This suggests a prevalence of the subcortical pathway in the right hemisphere for unconscious processing of emotional information (Ladavas et al., 1984; Cimatti et al., 1993; for a review, Gainotti, 2012). This view is also supported by neuroimaging evidence showing right amygdala activation to unseen fearful faces in a patient with cortical blindness (Burra et al., 2017) and to masked emotional stimuli in healthy participants (Costafreda et al., 2008).

## Conclusion

To conclude, the present findings provide evidence that lesions to the pulvinar prevent implicit visual processing of fear in hemianopic patients, supporting the hypothesis that the pulvinar nuclei of the thalamus play a considerable role in connecting unconscious threat-related visual information, from the SC to the amygdala. This is in line with the notion that the primate pulvinar might have evolved in part to assist in rapid threat detection and avoidance (Isbell, 2006), favoring adaptive defensive mechanisms.

## Author Contributions

CB and DB collected the data. CB and MP analyzed the data. CB, MP, DB, and EL designed the experiments and wrote the manuscript.

## Conflict of Interest Statement

The authors declare that the research was conducted in the absence of any commercial or financial relationships that could be construed as a potential conflict of interest.

## Footnotes

1 www.neurobs.com
